# The Influence of Yb Doping and Sintering Conditions on the Magnetocaloric and Mechanical Properties of EuS

**DOI:** 10.3390/molecules27175660

**Published:** 2022-09-02

**Authors:** Liang Li, Yuqi Chen, Junbao He, Aiguo Zhou

**Affiliations:** 1 School of Mechanical and Electrical Engineering, Nanyang Normal University, Nanyang 473061, China; 2 Jinguan Electric Co., Ltd., Nanyang 473000, China; 3 Henan Key Laboratory of Materials on Deep-Earth Engineering, School of Materials Science and Engineering, Henan Polytechnic University, Jiaozuo 454003, China; 4 School of Materials Science and Engineering, Shanghai Dian Ji University, Shanghai 201306, China

**Keywords:** polycrystalline EuS, magnetocaloric effect, magnetization, heat capacity

## Abstract

For this work, europium monosulfide (EuS) powders were prepared by sulfurizing Eu_2_O_3_ powder with CS_2_ gas. The synthesized EuS powders were sintered by SPS at temperatures in the 800–1600 °C range for 0.33–1 h at 50 MPa under vacuum conditions. The influences of Yb doping and sintering conditions on the magnetocaloric and mechanical properties of EuS were investigated systematically. An increase in sintering temperature caused the rise of lattice parameters of EuS, whereas Yb doping caused them to drop. SEM showed that the grain size of the EuS increased with sintering temperatures in the 1000–1400 °C range. Higher sintering temperatures can enlarge the magnetizability and saturation magnetization of EuS compact. On the contrary, Yb doping can weaken the magnetizability and saturation magnetization of EuS compact. All sintered polycrystalline EuS compacts had weaker thermomagnetic irreversibility and lower magnetic anisotropy.

## 1. Introduction

Magnetic refrigeration (MR) is a type of refrigeration technology that is based upon the magnetocaloric effect [1]. It has traditionally been researched for use in refrigeration at approximately room temperature [2]; however, current MR research has focused on an objective temperature of approximately 20 K using hydrogen liquefaction [3,4,5]. Liquidizing hydrogen is an efficient method for the application of hydrogen fuel [6,7,8,9]. A magnetic coolant required a phase transition near the liquefaction temperature of the liquid hydrogen [10,11]. Medium and heavy rare-earth compounds with high specific temperatures meet the practical requirements for magnetic refrigerant materials [10,12].

Recently, single-crystal ferromagnetic semiconductor cubic europium monosulfide (EuS) demonstrated substantial reversible magnetocaloric effects, with maximum magnetic entropy changes of 37 J·Kg^−1^·K^−1^ at 18.5 K for a magnetic field shift of 5 T; they can therefore be regarded as a possible magnetic refrigerant material for hydrogen liquefaction [13]. EuS with an NaCl structure is isotropic according to the result of single-crystal EuS [14,15]; thus, polycrystalline EuS can be used as a magnetic refrigerant material for hydrogen liquefaction.

Concerning the preparation of EuS, the current research is focused on the wet chemical synthesis of EuS nanoparticles [16,17] or the preparation of EuS films [15]. EuS nanoparticles have been synthesized by the thermolysis and colloidal routes and by the decomposition of single-source precursors [18]. Moreover, EuS nanoparticles from different precursors show different magnetic behaviors (ferromagnetic or paramagnetic) at 10 K [19,20,21]. The relationship between EuS magnetic properties and particle size and composition has been investigated based on the regulation of the preparation process of nanostructured EuS [22]. In our previous work, we achieved the controlled synthesis of EuS powder by a gas–solid chemical reduction method using nano-Eu_2_O_3_ powder with different morphological characteristics than the raw material [23].

The sintering process is a critical step in the industrial application of sulfides as energy storage materials. Regarding rare-earth sulfide sintering, the most common method is the growth of a single crystal of rare-earth sulfide by the closed-tube method [24,25]. Ebisu et al. systematically studied the growth of Ln_2_S_3_ (Nd_2_S_3_, Pr_2_S_3_, Tb_2_S_3_, Dy_2_S_3_ [26]) single crystals and their low-temperature physical properties. To overcome the high melting point of sulfide, the single-crystal growth process requires the addition of sintering aids. Hirai et al. synthesized CeS from a mixture of Ce_2_S_3_ and CeH_3_ as raw materials using a hot pressing method [27]. Yuan et al. sintered Gd_2_S_3_ and Ho_2_S_3_ using discharge plasma sintering and studied their thermoelectric properties [28].

As opposed to the sintering of Ln_2_S_3_, EuS having an NaCl-type structure causes it to crack easily during sintering. To systematically study the sintering properties, EuS compacts were sintered at temperatures in the 1273–1873 K range by spark plasma sintering. Sintering temperature, pressure, holding duration, heating rate, and other factors all have an impact on the performance of sintering EuS. Sintering temperature not only affects the chemical composition of the compact, but also influences the valence of Eu. The mechanical strength of sintered EuS cannot be guaranteed if the density of the sintered EuS bulk is too low. A longer keeping time can improve the uniformity of compact EuS and reduces the residual sulfur in the sulfurized EuS powder. The heating rate determines the formation of microcracks.

Doping is an effective way to improve the physical properties and electronic structure of EuS. Ferromagnetic coupling is weakened in the smaller europium sulfide nanoparticles, so increasing T_c_ with electron doping is of interest to study [22]. Gd-doped EuS nanocrystals resulted in enhancements of their magnetic properties and Curie temperature T_c_ [29]. The sodium-doped EuS (Eu_1−*x*_Na*_x_*S, x < 0.5) was synthesized via purposely introducing NaOH or NaCl to Eu_2_O_3_ [22]. Similarly, the T_c_ and paramagnetic Curie temperature, θ_p_, rose dramatically as a result of the doping concentration in Eu_1−*x*_Gd*_x_*S with gadolinium doping, although the data did not follow a smooth distribution [29]. As ytterbium (Yb) has similar variation characteristics, the focus has been predominantly on Yb. Understanding and regulating the variance of T_c_ requires research into the impact of Yb doping on the phase transition temperature of EuS.

In this study, polycrystalline EuS compacts were sintered in various sintering conditions and analyzed. We systematically measured the magnetic susceptibility, magnetization, and specific heat of polycrystalline EuS. The temperature and field dependences of the magnetic entropy change were calculated from the magnetization. The sintering process influences the grain growth of EuS; the effect of the grain size of EuS on the specific heat and phase transformation temperature was studied by sintering EuS at different sintering temperatures. The mechanical properties of EuS compacts were also investigated.

## 2. Results

### 2.1. Sintering of EuS and Yb-Doped EuS

**Figure 1:**
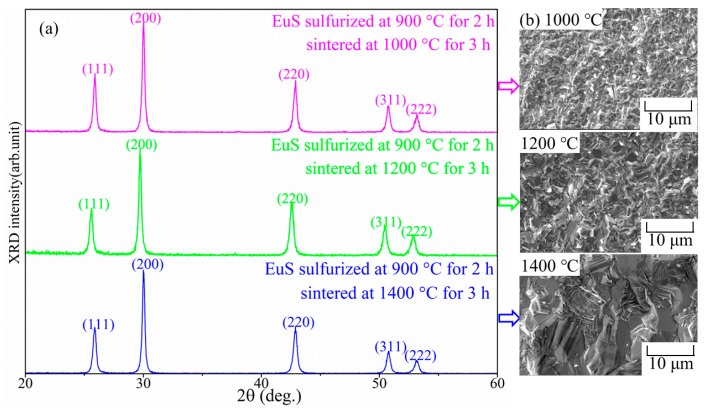
XRD patterns (**left side**, **a**) and SEM images (**right side**, **b**) of EuS bulks prepared by SPS.

Figure 1a shows typical XRD patterns of synthetics by spark plasma sintering. For all sintering temperature ranges, single-phase EuS compacts might have been formed; there was no distinctive peak for Eu_2_O_2_S. On the other hand, characteristic peaks of EuS tended to have small angles following an increase in sintering temperature, indicating that the lattice constants increased with a rise in sintering temperature.

**Figure 2:**
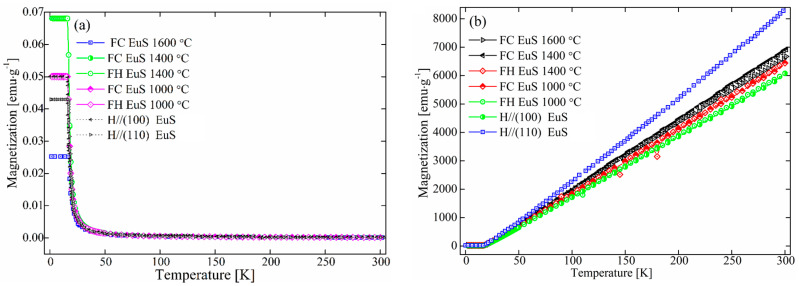
The relationship between FH and FC magnetization of the prepared EuS and temperature: (**a**) the temperature dependence of magnetization M(T); (**b**) the inverse susceptibility curve, 1/χ, for sintered polycrystalline EuS; the magnetic data of single-crystal EuS along (100) and (110) directions [13] were added to compare the difference between single-crystal and polycrystalline EuS magnetic data.

**Figure 3:**
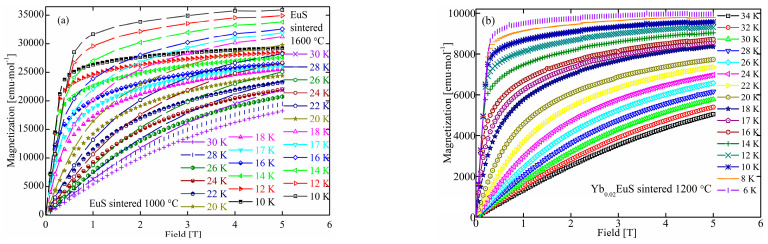
Magnetization of polycrystalline undoped EuS (**a**) and Yb-doped EuS (**b**) as a function of the field.

Table 1 shows the lattice constants of EuS and Yb-doped EuS synthesized under different sintering conditions. The lattice constant of compact EuS increased with the increase in temperature. The lattice constant of Yb-doped EuS decreased after doping. The change in sintering temperature not only affected the grain size (refer to Figure 1b for SEM results), but also affected the mixed valence state of the Eu, which could be explained by magnetic data (Figure 2 and Figure 3).

SEM micrographs of cross-sections of the synthesized EuS sections with the different sintered temperatures for 3 h are displayed in Figure 1b. The visible grain growth of EuS could be observed with increasing sintering temperature. The structure of the EuS that was sintered by SPS was dense and homogeneous. Some brittle fractures could be observed at the grain boundary, indicating that the cleavage plane should be attributed to the crack in the sintered EuS.

### 2.2. Magnetizations of EuS and Yb-Doped EuS Bulks

The temperature dependences of magnetization M(T) for sintered polycrystalline EuS bulks were measured under a 100-Oe magnetic field and the magnetic data of single-crystal EuS along (100) and (110) directions [13] were added for comparison as shown in Figure 2. Temperature-dependent magnetizations were examined in cooling (FC) and heating (FH) states under the presence of an applied magnetic field. Figure 2 shows that all sintered polycrystalline EuS compacts had almost the same FC and FH curves throughout the entire temperature range. There were weaker or smaller temperature-dependent irreversibilities and magnetic asymmetries. The paramagnetic to ferromagnetic transition for polycrystalline EuS occurred at 18.2 K.

Table 2 lists the magnetization at 2 K, Curie temperature (T_c_), and Curie parameters of EuS sintered at temperatures in the 1000–1600 °C range. The sintering temperature had an effect on the magnetization at low temperatures. The EuS sintered at 1400 °C had the maximum magnetization value. The polycrystalline EuS prepared by spark plasma sintering had a similar magnetization value as the reported single-crystal EuS [13]. The magnetization of EuS sintered at 1600 °C was the lowest. The main factors that affected magnetization were the grain size and impurity content of the EuS.

At temperatures above the T_c_, the inverse susceptibility curve, 1/χ, was linear and conformed to the modified Curie–Wiess law, χ(T) = χ_0_ + C/(T − θ_p_), where C stands for the Curie constant, θ_p_ represents the paramagnetic Curie temperature, and χ_0_ is the temperature-independent term. The M(T) function follows the Curie–Weiss law, and the calculations of the paramagnetic Curie temperature θ_P_ also showed this.

The single-crystal EuS Curie temperature (16.8 K) was comparable with that of the sintered EuS. The Curie temperature gradually increased as the sintering temperature rose. Due to substantial indirect interaction between the impurity electron and the localized 4f states, Curie temperatures as a function of electron concentration in ferromagnetic EuS crystals rise quickly [30]. These results were consistent with the reported Curie temperature (18 K) of EuS [31].

The least square fits from T_c_ to 300 K yielded effective moments (μ_eff_) of 8.06 μ_B_/Eu and 8.26 μ_B_/Eu for EuS prepared at 1000 °C and 1400 °C, respectively. The μ_eff_ of sintered EuS was slightly larger than 7.2 μ_B_/Eu for EuS sintered at 1600 °C and 7.9 μ_B_/Eu for single-crystal EuS. Compared to single-crystal EuS, polycrystalline EuS has a lower magnetization. The occupied 4f electrons in the majority spin channel define the μ_eff_ of Eu, which is equal to 7.9 μ_B_, in theory. The calculated magnetic moment, μ_eff_/Eu, was 6.93 μB and μ_eff_/Eu was found to be 6.9 μ_B_ throughout the EuS/Bi_2_Se_3_ film [14]. Similar to the EuS/InAs film, the magnetic part was mostly concentrated in the EuS thin film with a restriction of the Eu moment in the EuS layer closest to the InAs [32].

Figure 3a depicts the connection between temperature or magnetic field and isothermal magnetism M(H, T)/M(H, B) of sintered EuS. As seen in Figure 3, M(H, T) showed ferromagnetic behavior at low temperatures; however, polycrystalline EuS sintered at 1600 °C had larger values than that of EuS prepared at 1000 °C under a given field and temperature, which was consistent with the grain size of EuS (as shown in Figure 1b). Higher sintering temperatures led to a larger grain size in EuS. Similar results were observed in EuS nanocrystals (NCs, with an average size of 44 nm) and nanorods (NDs, with an average size of 7.5 nm). The EuS NCs had much stronger magnetizations than those of the EuS NDs [33]. Compared with the magnetization of single-crystal EuS following the orientations in (100) and (110) as shown in Figure 2, both magnetizations of EuS sintered at 1000 °C (less than 160 emu/g) and 1600 °C (less than 190 emu/g) were weaker than that of single-crystal EuS (above 200 emu/g). This might be explained by the presence of impurities left behind in the EuS powders after the sulfurization process of Eu_2_O_3_, such as residual carbon or oxygen atoms.

At low temperatures, the saturation point of the magnetization was achieved before 1 T. After the temperature rose, this pattern weakened. With an increase in the magnetic field over 22 K, the magnetization rose almost linearly. For a certain field, magnetization decreased as the temperature rose.

Figure 3b displays the magnetization of Yb-doped EuS as a function of temperature at different magnetic fields. Compared with undoped EuS compacts, a small amount of Yb doping had little influence on the relationship between magnetization and temperature/magnetic field (similar to what is shown in Figure 3a). However, the saturation magnetization of Yb-doped EuS obviously decreased. It was presumed that the coupling of 4f^14^ local electrons of Yb^2+^ and 4f^7^ electronic layer of Eu^2+^ led to the enhancement of electron local hybridization and the decrease in magnetization. M tended to saturate at about 1.0 × 10^4^ emu/moL at low temperatures.

**Figure 4:**
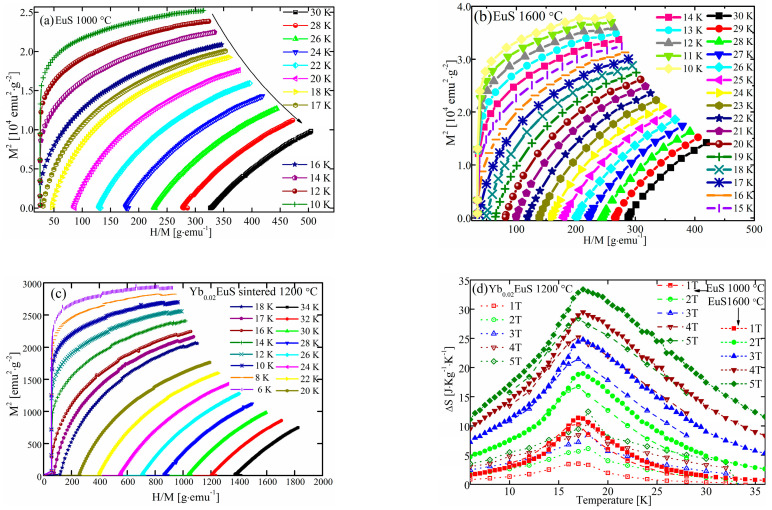
The Arrott plots (M^2^ versus H/M) of polycrystalline undoped EuS sintered at 1000 °C (**a**) and 1600 °C (**b**); Yb-doped EuS (**c**); and the relationship between temperature and magnetic entropy variations of sintered EuS (**d**).

The inflection point that appears in the Arrott plot for the polycrystalline EuS in Figure 4 revealed that there was a magnetic change from disordered paramagnetic to the ordered ferromagnetic arrangement at a temperature that exceeded the Tc. The Arrott plot’s positive slope (M^2^ vs. H/M) indicated that the phase change was of second order.

A magnetic entropy change, ΔS, can be obtained using the Maxwell relation: ΔS(T, H) = $\int_{0}^{H}{\left(\frac{\partial M}{\partial T}\right)}_{H}\mathrm{dH}$. Figure 4d shows the variation in magnetic entropy of polycrystalline EuS and Yb-doped EuS and its relation to temperature for various magnetic fields. The total amount of ΔS for EuS sintered at 1600 °C grew first before starting to decline when an upper limit was reached. Additionally, the maximum ΔS was obtained at a slightly higher temperature of 17.97 K for ΔH = 5 T than it was at 17.47 K for ΔH = 1 T. Above the Curie temperature, a sizable MCE was provided. The peak ΔS values were 2.02, 3.57, and 6.32 J/mol/K for applied fields of 1, 2, and 5 T, respectively.

The entropy changes in EuS sintered at 1600 °C were larger than those of EuS sintered at 1000 °C and Yb-doped EuS. The fact that the reported ΔS value for polycrystalline EuS prepared by spark plasma sintering was comparable to that of single-crystal EuS generated via a more difficult procedure must be emphasized. Rare-earth monosulfide or sesquisulfide crystals seldom exhibit a ΔS with such a high magnitude.

### 2.3. Specific Heat of EuS and Yb-Doped EuS

**Figure 5:**
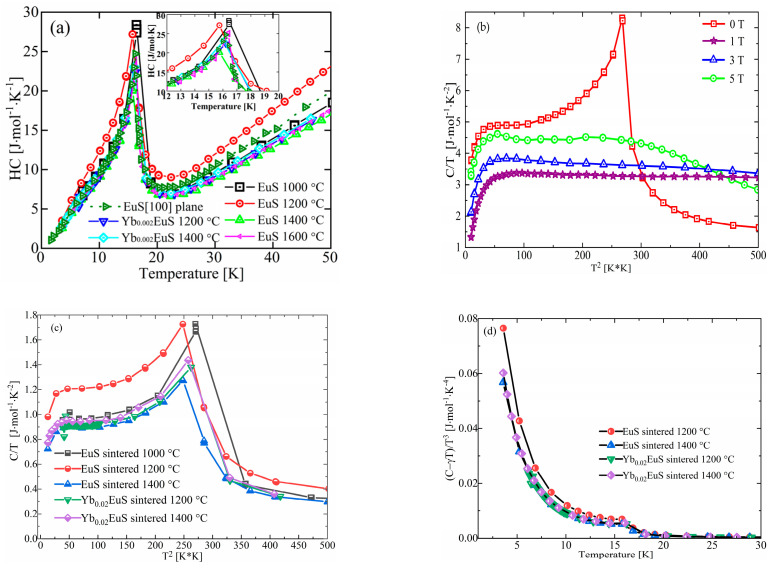
Temperature dependences of the specific heat, C(T), of Yb-doped EuS and undoped EuS [13] (**a**); plots of C/T vs. T^2^ for EuS (**b**) and Yb-doped EuS (**c**); the temperature dependence of (C − γT)/T^3^ (**d**).

The investigations of the temperature dependences of the specific heat C(T) of Yb-doped and undoped EuS were conducted in the absence of magnetic fields, as shown in Figure 5a. A significant peak can be seen on the C(T) curve at about 16.4 K in the zero field. For polycrystalline EuS, as the sintering temperature increased from 1000 °C to 1400 °C, the characteristic peak decreased from 16.4 K to 15.7 K. The transformation temperature shifted to a small angle and the specific heat decreased as the sintering temperature rose. The change in characteristic peak of the Yb-doped EuS was not obvious.

To estimate the magnetic entropy change based on the Maxwell relation and compute the adiabatic temperature change ΔT_ad_, the C/T vs. T^2^ of EuS sintered at 1600 °C was determined in magnetic fields of 0 and 5 T. The plots of C/T vs. T^2^ of EuS are shown in Figure 5b. At about 16.4 K, the C/T vs. T^2^ of EuS displayed a significant peak at the zero field. The peak weakened and disappeared as the magnetic field grew stronger.

The plots of C/T vs. T^2^ of EuS and Yb-doped EuS sintered at temperatures in the 1000–1400 °C range are shown in Figure 5c. Similar to EuS sintered at 1600 °C, the peak of phase transition of EuS occurred near 16.8 K at the zero magnetic field. With increasing sintering temperature and Yb doping, the Curie temperature of the phase transition of EuS decreased. As the antiferromagnetic transition takes place at low temperatures, it was challenging to estimate the Sommerfeld coefficient of the electronic specific heat, γ. By linearly fitting experimental data at 10–20 K, it was feasible to estimate the electronic specific heat parameter, γ. The findings unambiguously demonstrated that for all EuS and Yb-doped EuS, the intercept γ was positive and nonzero; this demonstrated the presence of some conduction electrons, which is similar to that of GdS [34] and YbS [35]. The following formula estimates the Debye temperature, θ_D_:

θ_D_ = (12π^4^R_g_n/5β)^1/3^ (1)

where R_g_ represents the gas constant, and the unit for the lattice term β is J∙mol^−1^∙K^−4^.

Using the Debye model for EuS and Yb-doped EuS, the temperature dependency of the lattice contribution, C_lat_, was assessed. The following equation can be used to express the C_lat_:

(2) $$C_{\mathrm{lat}}{=9N}_{A}\delta k_{B}{\left(\frac{T}{\theta_{D}}\right)}^{3}\int_{0}^{\theta_{D}/T}\frac{z^{4}e^{z}}{{{(e}^{z}-1)}^{2}}\mathrm{dz}$$

where δ is the quantity of atoms in a formula unit. The Boltzman’s constant, k_B_, and the Avogadro constant, N_A_, are both used. The temperature dependency of (C − γT)/T^3^ is shown in Figure 5d. The θ_D_ for EuS sintered at temperatures in the 1000–1400 °C range was different. The coefficients for the T and T^3^ terms of the specified temperature dependency of the heat capacity can be obtained from Figure 5d. For polycrystalline EuS, the θ_D_ increased from 175 K to 204 K following a rise in the sintering temperature. These values were close to the reported θ_D_ of 208 K [36]. Yb doping had a weaker influence on the θ_D_ of EuS.

### 2.4. The Influence of Sintering Conditions on the Mechanical Properties of EuS

**Figure 6:**
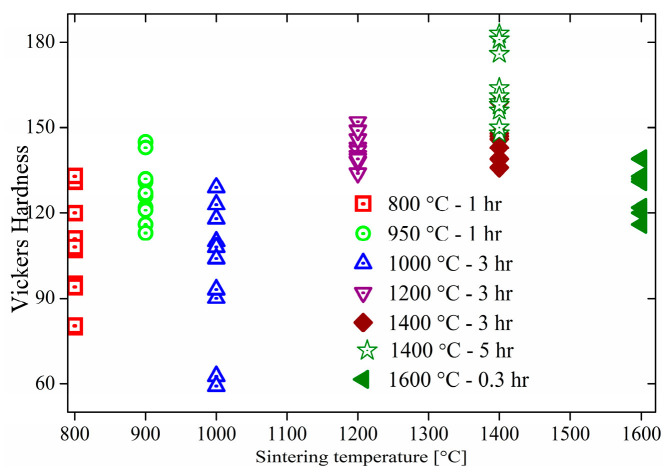
The hardness of sintered EuS compacts.

Mechanical strength, hardness, and density are important for the ceramic forming process and the possibility of industrial application. An optical photograph of EuS is shown in Figure S1. Figure 6 shows the hardnesses of EuS compacts sintered at different conditions. The hardness of EuS enlarged with a rise in sintering temperature and duration. The hardness of sintered EuS was highest at 1400 °C for 5 h, but sintered EuS bulk was easy to break after sintering. Complete compact EuS could be obtained after sintering at 1600 °C (diameter 20 mm × thickness 4.5 mm).

## 3. Discussion

**Figure 7:**
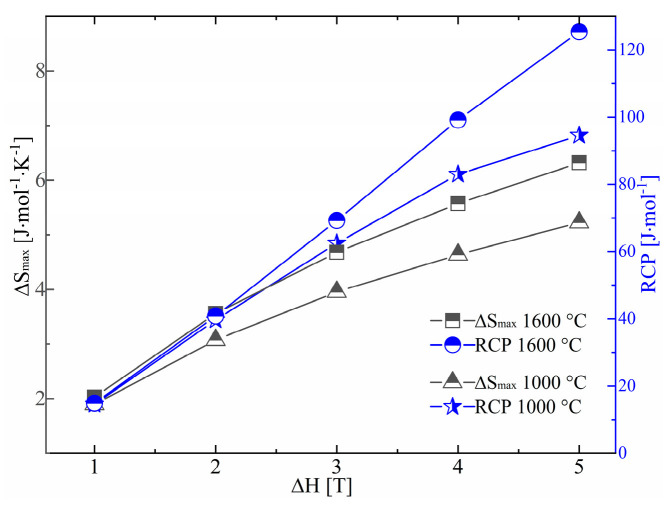
The ΔS_max_ and relative cooling power of EuS compacts sintered at 1000 °C and 1600 °C.

An essential factor of a magnetic refrigerant material is its relative cooling power (RCP), which is typically given as RCP, where S_max_ represents the peak of ΔS from Figure 6 and δT_FWHM_ stands for the effective length at half of the maximum of the matching ΔS. The aforementioned equation was used to calculate the ΔS_max_ and RCP, as illustrated in Figure 7. The very large values of ΔS_max_ and RCP, which grew monotonically with increasing ΔH, showed that polycrystalline EuS exhibited exceptional magnetic refrigeration properties. The ΔS_max_ for EuS was larger than that of ErFeSi (5.8 J/mol/K) [10], PrNi (1.2 J/mol/K) [37], DyNi_2_ (5.96 J/mol/K) [38], and HoN(5.04 J/mol/K) [39] for applied fields of 5 T. For EuS prepared at 1000 °C and 1600 °C, the RCP levels were 94.6 and 125.4 J/mol at ΔH = 5 T, respectively. The RCP for polycrystalline EuS prepared by spark plasma sintering was a little lower than that for a single crystal (143.94 J/mol at ΔH = 5 T). The RCP for polycrystalline EuS was superior to that of ErFeSi (117.5 J/mol) [10], PrNi (12.1 J/mol/K) [37], DyNi_2_ (123.3 J/mol/K) [38], and HoN(100.9 J/mol/K) [39] for applied fields of 5 T.

Heat capacities for polycrystalline and single-crystal EuS under various magnetic fields were examined, as illustrated in Figure S3, to further understand the differences in the magnetocaloric characteristics between both types of EuS. At temperatures below 20 K without magnetic fields, the specific heat values were nearly identical. In comparison to single-crystal EuS, the paramagnetic polycrystalline EuS phase had a somewhat lower heat capacity.

Rare-earth element Yb-doped EuS had the advantage of being able to easily form a solid solution; however, its effect on magnetic modulation was not obvious, which might be related to the electronic structure of Yb 4f^14^. The doping of magnetic transition metal elements Fe and Co will be considered in the future. SEM images and EDS analysis of Yb-doped EuS are shown in Figure S2. Compared with the results of EuS in Figure 2, Yb doping with a low melting point (819 °C) could improve the denseness of EuS bulk.

## 4. Materials and Methods

The preparation process of EuS powder was described in our previous report [40]. Synthetic EuS powders were directly sintered at temperatures in the 800–1600 °C range for 0.33–5 h via spark plasma sintering, at less than 50 MPa (Model SPS-511L, Sumitomo Coal Mining Co., Ltd., Tokyo, Japan). A constant heating rate of 0.42 K·s^−1^ was adopted. The vacuum was less than 7 × 10^−3^ Pa during sintering. Cold pressing was performed on all samples with a 25 MPa uniaxial applied stress.

The synthesized compounds were identified by X-ray diffraction (XRD), Model Rint-Ultima+, Rigaku Corp., Tokyo, Japan, with monochromatic Cu-Kα radiation at an accelerating voltage of 40 kV and a filament electric current of 20 mA. Scanning electron microscopy was used to characterize the morphology of the compacts (SEM, JSM-5310LV, JEOL Ltd., Tokyo, Japan).

A superconducting quantum interference device (SQUID, Quantum Design, San Diego, CA, USA ) magnetometer was used to detect magnetization as a function of temperature (between 2 and 300 K) and magnetic field (between 0 and 5 T). Utilizing the physical properties of the measuring equipment, the thermal relaxation approach was used to detect specific heat in the temperature range between 2 and 50 K (PPMS, Quantum Design).

## 5. Conclusions

EuS and Yb-doped EuS compacts were sintered at temperatures in the 800–1600 °C range by SPS. The lattice parameters of undoped EuS increased with sintering temperature, while Yb-doped EuS compacts changed without regularity. The grains of EuS grew obviously with an increase in temperature. At the same magnetic field strength, the induction magnetization and saturation magnetization increased with an increase in EuS grain size. On the contrary, Yb doping could decrease the induction magnetization and saturation magnetization of EuS. Magnetic entropy changes and heat capacity displayed similar trends. It should be noted that the current study demonstrated a novel material approach for hydrogen liquefaction. Finally, it is hoped that the Curie temperature of EuS will be adjusted to around 20 K by element doping in the future.

## Figures and Tables

**Table 1:** Sulfurization conditions, sintering conditions, and lattice parameters of sintered EuS and Yb-doped EuS.

Sample	Sulf. Condition	Lattice Constant
EuS	1000 °C	5.959182
EuS	1200 °C	5.969099
EuS	1400 °C	5.979182
Yb_0.02_EuS	1000 °C	5.971716
Yb_0.02_EuS	1200 °C	5.962835
Yb_0.02_EuS	1400 °C	5.971736

**Table 2:** Magnetization at 2 K, Curie temperature (T_c_), and Curie parameters of EuS sintered at temperatures in the 1000–1600 °C range.

Sintering Temperature	M(2 K) [emu/g]	T_c_ [K]	C	θ_p_ [K]	μ_eff_ (μB)
1000 °C	0.0500	18.0	8.13	18.8	8.06
1400 °C	0.0681	18.0	8.54	19.6	8.26
1600 °C	0.0001	18.2	6.48	17.0	7.20
Single-crystal EuS (100) plane [13]	0.0430	19.0	7.58	17.2	7.79
Single-crystal EuS (110) plane [13]	0.0500	19.0		16.9	7.91

## Data Availability

The data presented in this study are available on request from the corresponding author.

## Supplementary Materials

The following supporting information can be downloaded at: https://www.mdpi.com/article/10.3390/molecules27175660/s1, Figure S1. The optical photograph of EuS; Figure S2. SEM images and EDS analysis of Yb doped EuS; Figure S3. Comparison of heat capacity for polycrystalline and single crystal EuS under zero field (a); 1T (b); 3 T (c); and 5 T (d).
