# Supplementary material for: The Influence of Yb Doping and Sintering Conditions on the Magnetocaloric and Mechanical Properties of EuS
Source: Molecules. 2022 Sep 2;27(17):5660. doi: 10.3390/molecules27175660 (PMC9457925; doi:10.3390/molecules27175660)
Supplement: Supplementary file 1 [file molecules-27-05660-s001.zip › molecules-1889340-supplementary.pdf]

molecules

Supplementary

Influence of Yb doping and sintering conditions on magnetocaloric and mechanical properties of EuS

Liang Li [1,2,3,*], Yuqi Chen [4], Junbao He [1], and Aiguo Zhou [3,*]

1 School of Mechanical and Electrical Engineering, Nanyang Normal University, Nanyang 473061, China
2 Jinguan Electric Co., Ltd., Nanyang 473000, China
3 Henan Key Laboratory of Materials on Deep-Earth Engineering, School of Materials Science and Engineering, Henan Polytechnic University, Jiaozuo 454003, China
4 School of Materials Science and Engineering, Shanghai Dian Ji University, Shanghai 201306, China
* Correspondence: liliangjdgc@nynu.edu.cn; zhouag@hpu.edu.cn；Tel.: (+86-037763513077)

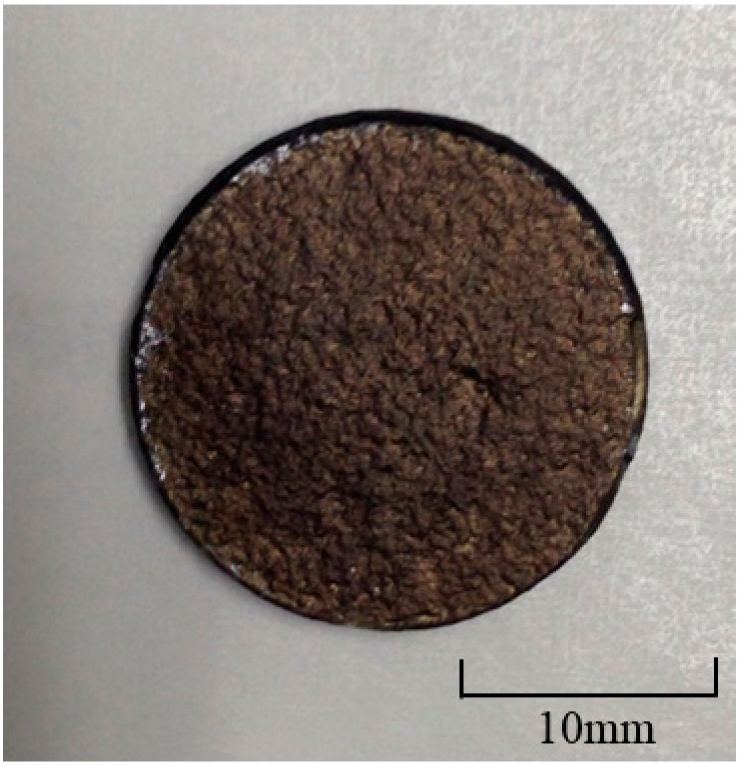

Figure S1. The optical photograph of EuS.

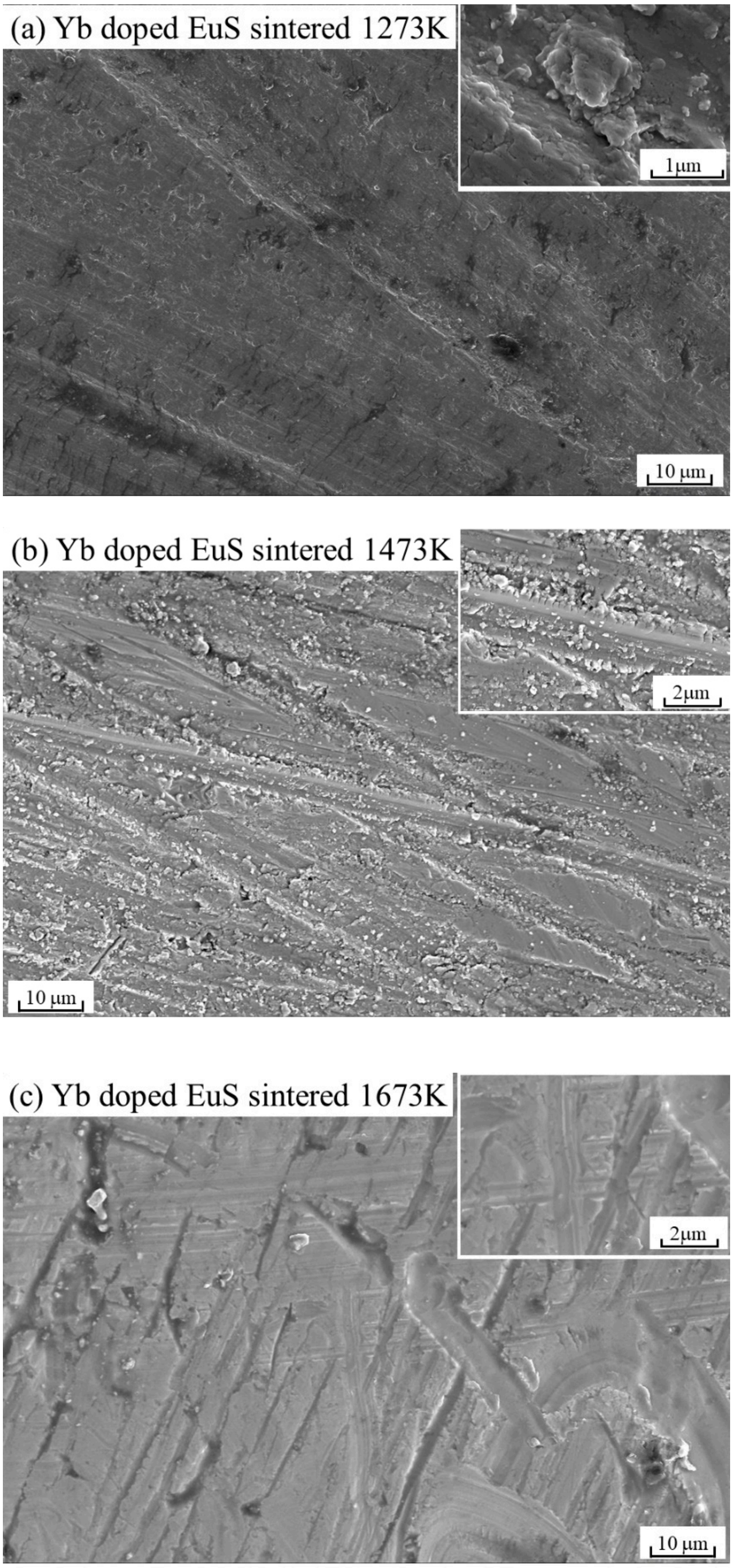
(a) Yb doped EuS sintered 1273K
1μm
10 μm
(b) Yb doped EuS sintered 1473K
2μm
10 μm
(c) Yb doped EuS sintered 1673K
2μm
10 μm

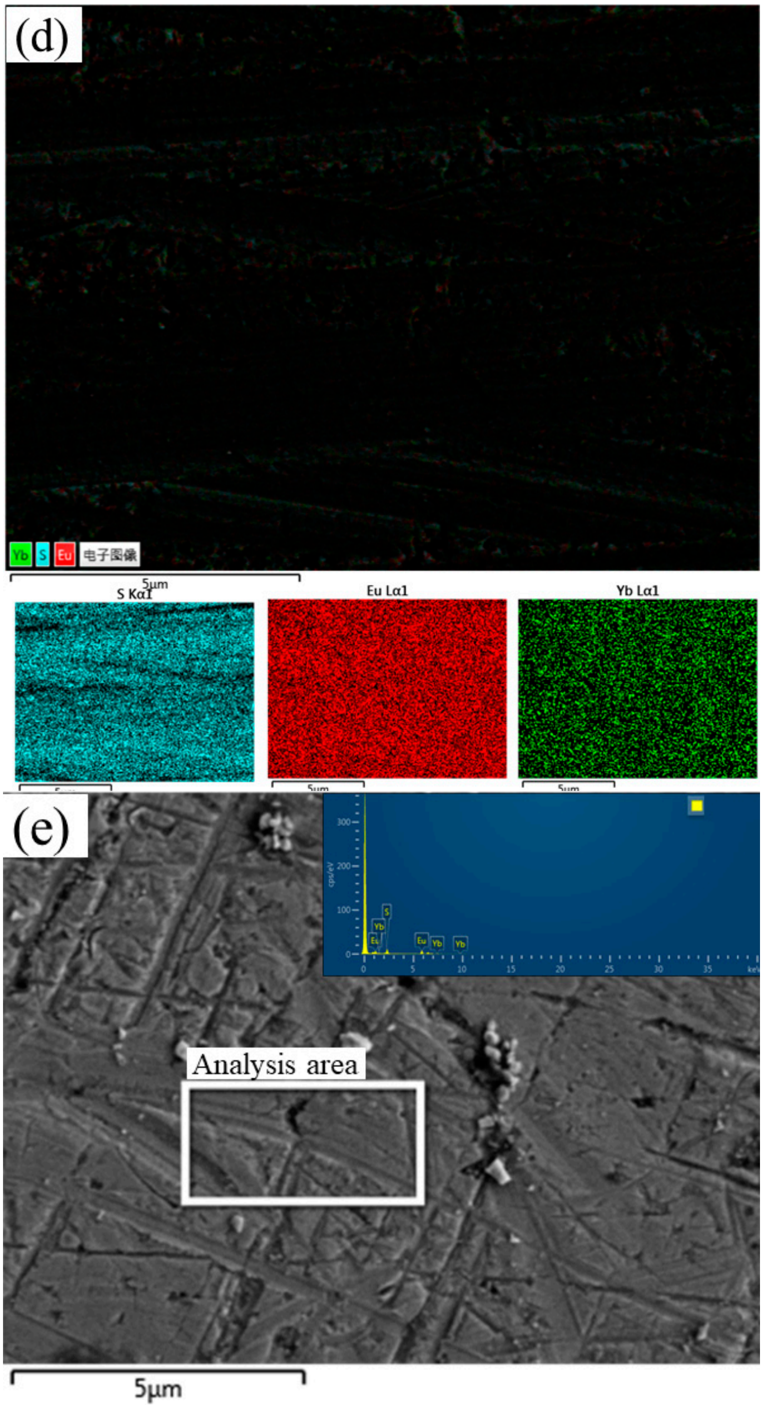

Figure S2. SEM images and EDS analysis of Yb doped EuS.

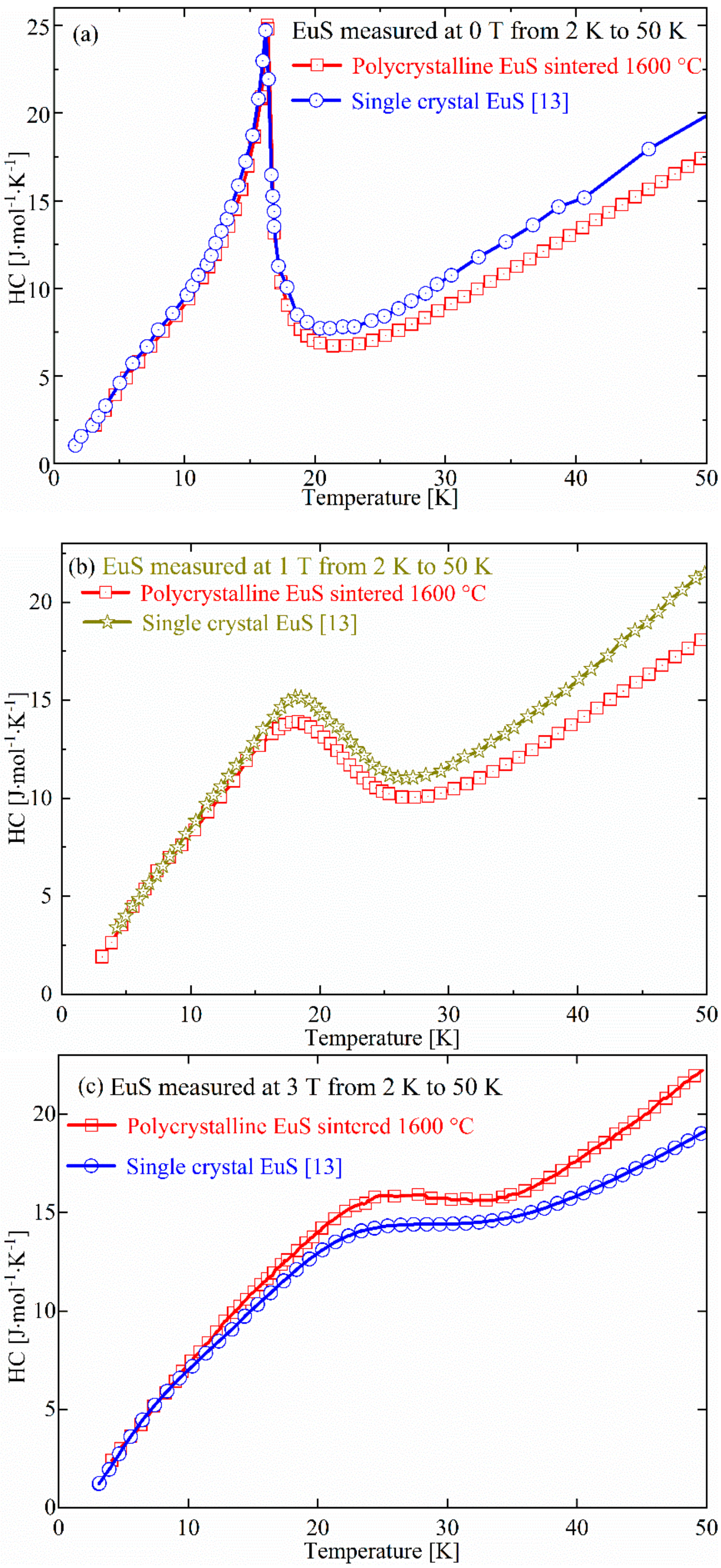
(a)
EuS measured at 0 T from 2 K to 50 K
Polycrystalline EuS sintered 1600 °C
Single crystal EuS [13]
HC [J·mol-1·K-1]
Temperature [K]
(b) EuS measured at 1 T from 2 K to 50 K
Polycrystalline EuS sintered 1600 °C
Single crystal EuS [13]
HC [J·mol-1·K-1]
Temperature [K]
(c) EuS measured at 3 T from 2 K to 50 K
Polycrystalline EuS sintered 1600 °C
Single crystal EuS [13]
HC [J·mol-1·K-1]
Temperature [K]

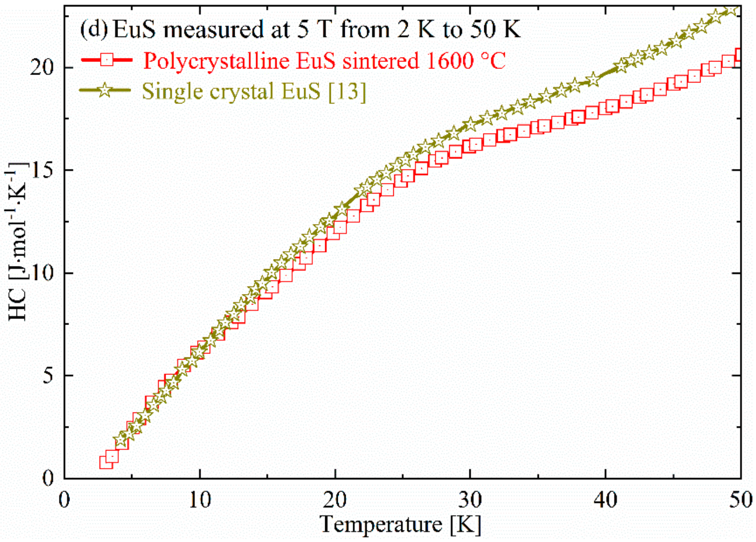

Figure S3. Comparison of heat capacity for polycrystalline and single crystal EuS under zero field (**a**); 1T (**b**); 3 T (**c**); and 5 T (**d**); the heat capacity data of single-crystal EuS [13] were added to compare the difference between single-crystal and polycrystalline EuS.
